# Mimickers of Intestinal Tuberculosis: Could this be Crohn’s Disease? An Unsolved Enigma

**DOI:** 10.4103/1319-3767.77236

**Published:** 2011

**Authors:** Preethi G. K. Venkatesh, Udayakumar Navaneethan

**Affiliations:** Digestive Disease Institute, The Cleveland Clinic, Cleveland, Ohio,

The incidence of intestinal tuberculosis (TB) is increasing in developed countries owing to human immunodeficiency virus infection,[1] while the incidence of Crohn’s disease (CD) in TB endemic areas also appears to be increasing.[2] Both diseases have a predilection for the small bowel, particularly the terminal ileum, although any part of the gastrointestinal tract may be affected. This poses considerable diagnostic dilemma as both intestinal TB and CD are chronic granulomatous disorders[3] with similarities that make the differentiation between these two entities very difficult. Intestinal TB has been misdiagnosed as CD for as long as seven years before the right diagnosis was reached.[4] In a Chinese study, up to 65 % of CD had been misdiagnosed as intestinal TB.[5] In addition to the above conditions, carcinoma, amebiasis, enteric fever or Yersinia infection can mimic symptoms of intestinal TB and cause diagnostic confusion.[6,7]

In this issue of the journal, the authors report a series of patients with intestinal TB from India.[8] They studied 100 patients who underwent intestinal resection and included 22 patients with clinical suspicion of intestinal TB. On final analysis, only six cases were proved histologically as TB, while nine patients were diagnosed as ischemic enteritis, four patients as chronic nonspecific enteritis (presented clinically as acute intestinal obstruction), one patient as cecal carcinoma, one patient as CD and one patient as intussusception. The authors highlighted that a number of other intestinal diseases can masquerade as TB.

This study from India is significant in the sense that it highlights the varied clinical presentation of other intestinal pathologies that may present with symptoms and/or signs of intestinal TB. It is not surprising that in a TB endemic country like India, patients presenting with strictures, perforation and/or right iliac fossa mass with abdominal pain, TB is in the top of the differential diagnosis. However, most patients with suspected TB did not turn out to have the disease. It is not surprising given that TB is often confused with CD and carcinoma.[6] It is interesting that there were nine patients with ischemic enteritis which were diagnosed at pathology. Ischemic enteritis occurs due to interruption or significant decrease of the arterial blood flow to the small intestine. Elderly patients are most often affected, while younger patients, especially those with diabetes, lupus erythematosus or sickle-cell anemia, may also present with ischemic enteritis.[9] However, a definite diagnosis of the disease is usually established after histopathological results of the resected bowel segment have been obtained. We unfortunately do not have information on the clinical presentation or imaging on those patients and hence it is very difficult to understand the clinical setting in which the diagnosis of intestinal TB was entertained. There are, however, reports of ischemic enteritis masquerading as CD. Hence, it is not surprising that TB was in the differential diagnosis in these patients.

Abdominal tenderness with mass in the right iliac fossa was the most common presenting symptom (100%) of TB in this series. The authors confidently diagnosed TB based on the presence of caseous granulomas on histopathology.[8] The authors also mention that they had diagnosed four patients with chronic non-specific enteritis, mentioning that these patients did not have any granulomas suspicious for either TB or CD.[8] However, granulomas occur in less than 50% of patients with CD or TB[10] and hence the whole clinical picture along with biopsy and long-term follow up to understand disease evolution may ultimately help in confirming the diagnosis of non-specific enteritis.

The diagnostic confusion in approaching patients with right iliac fossa problems with suspected TB and CD is highlighted by this case series. The ileocecal region is commonly involved in TB secondary to the high concentration of lymphoid aggregates in this area and possibly also due to the prolonged contact between the bacilli and mucosa.[6] Similarly in CD, ileocolonic lesions are seen predominantly.[6] The clinical presentation of both TB and CD are similar, except that patients with CD are more likely to be younger, present with aphthoid ulcerations, perianal disease, enteric fistulae and extraintestinal manifestations, bleeding per rectum, diarrhea and shorter duration of symptoms.[6] Similarly endoscopic features described in patients with TB and CD is suggested to help in the differential diagnosis; however, most patients have nonspecific ulcers on ileocecal valve and cecum without typical features. TB most commonly presents with transverse or linear ulcers, nodules, a deformed ileocecal valve and cecum, presence of inflammatory polyps, and multiple fibrous bands arranged in a haphazard fashion. CD on the other hand presents with segmental longitudinal ulcers with a cobble stone appearance, stricture, perianal lesion and pseudo polyps. Computerized tomography imaging with evidence of asymmetric bowel thickening and necrotic lymph nodes is more suggestive of intestinal TB.[6] The use of polymerase chain reaction (PCR) has been found to have a high accuracy for diagnosing intestinal TB with a specificity of up to 95 % and an accuracy of 82.6 %.[11] Further studies exploring PCR in fecal samples and in situ PCR, where the targeted sequence is amplified within intact cells, are being studied and look very promising.[6]

To conclude, intestinal TB is a great mimicker of other diseases and a high clinical suspicion along with other supporting clinical, endoscopic, imaging and histopathologic features is required to accurately diagnose and treat patients.
